# Fluid removal in critically ill patients during hemodialysis: is there a role for functional hemodynamic monitoring?

**DOI:** 10.1186/cc10178

**Published:** 2011-06-22

**Authors:** RH Passos, PB Batista

**Affiliations:** 1 Hospital São Rafael, Salvador - BA, Brazil

## Introduction

Renal replacement therapy is frequently required in critically ill patients with acute kidney injury. With intermittent hemodialysis, large volumes of fluid need to be removed over a relatively short period of time, jeopardizing hemodynamic stability in already hemodynamically compromised patients. Established methods of dry weight estimation are not practical in critical care and the estimation of excess body fluid removable by hemodialysis constitutes a particular change in these patients. Dynamic parameters of fluid responsiveness are increasingly being used to guide fluid therapy in critical care, but their suitability to monitor fluid removal with hemodialysis is not known.

## Objective

The aim of our study was to analyze changes in a dynamic parameter of fluid responsiveness (pulse pressure variation) in critically ill patients submitted to intermittent hemodialysis.

## Methods

Changes in pulse pressure variation, central venous pressure, median arterial pressure, and cardiac index were analyzed every hour over intermittent hemodynamics using a minimally hemodynamic monitoring device (LIDCO plus) in 28 mechanically ventilated patients. Additional measurements of lactate and central venous saturation were measured at the same time.

## Results

Median dialysis duration was 4.5 hours, and a median of 2,900 ml fluid was removed. There were 102 hypotensive episodes. The median arterial blood pressure was 72 mmHg. Median CVP was 16 ± 6 and pulse pressure variation was 9 ± 6 just before hemodialysis. There was a significant increase in the pulse pressure variation over the dialysis treatment (15 ± 4) and a decrease in the CVP value (13 ± 6). Comparing the group of patients already fluid responsive (ΔPp >13%) just before the start of hemodialysis with the group nonfluid reponsive (ΔPp <13%), the median values of lactate (2.1 × 1.9, *P *= 0.78) and central venous saturation (0.74 × 0.72, *P *= 0.94) were not significantly different, but at the end of the procedure a significant difference in lactate was observed (4.2 × 2.5, *P *< 0.2).

## Conclusion

In our study the rate of ultrafiltration during hemodialysis was reflected by the changes in the pulse pressure variation. In patients already fluid responsive (ΔPp >13%) just before hemodialysis, the impact of fluid removal at the end of the procedure in perfusion parameters was significantly higher. Dynamic parameters of volemia could be useful to guide fluid removal and avoid hypoperfusion in acute renal failure patients mechanically ventilated during hemodialysis treatment.

